# Genetics and Genomics of Cotton Leaf Curl Disease, Its Viral Causal Agents and Whitefly Vector: A Way Forward to Sustain Cotton Fiber Security

**DOI:** 10.3389/fpls.2017.01157

**Published:** 2017-07-04

**Authors:** Mehboob-ur- Rahman, Ali Q. Khan, Zainab Rahmat, Muhammad A. Iqbal, Yusuf Zafar

**Affiliations:** ^1^National Institute for Biotechnology and Genetic EngineeringFaisalabad, Pakistan; ^2^Pakistan Agricultural Research CouncilIslamabad, Pakistan

**Keywords:** begomoviruses, CLCuD, genome editing, introgression breeding, marker-assisted breeding

## Abstract

Cotton leaf curl disease (CLCuD) after its first epidemic in 1912 in Nigeria, has spread to different cotton growing countries including United States, Pakistan, India, and China. The disease is of viral origin—transmitted by the whitefly *Bemisia tabaci*, which is difficult to control because of the prevalence of multiple virulent viral strains or related species. The problem is further complicated as the CLCuD causing virus complex has a higher recombination rate. The availability of alternate host crops like tomato, okra, etc., and practicing mixed type farming system have further exaggerated the situation by adding synergy to the evolution of new viral strains and vectors. Efforts to control this disease using host plant resistance remained successful using two gene based-resistance that was broken by the evolution of new resistance breaking strain called Burewala virus. Development of transgenic cotton using both pathogen and non-pathogenic derived approaches are in progress. In future, screening for new forms of host resistance, use of DNA markers for the rapid incorporation of resistance into adapted cultivars overlaid with transgenics and using genome editing by CRISPR/Cas system would be instrumental in adding multiple layers of defense to control the disease—thus cotton fiber production will be sustained.

## Introduction

**FIGURE 1:**
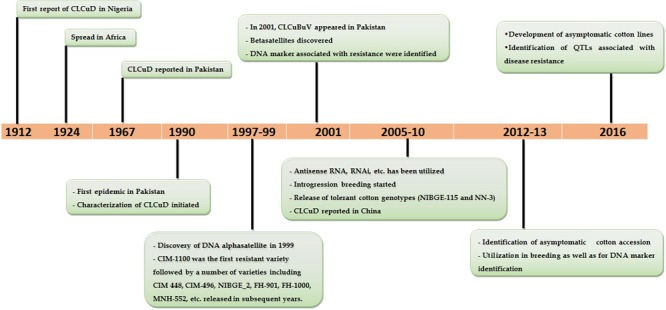
Important landmarks in the history of cotton leaf curl disease (CLCuD) (from first emergence till to date).

Cultivation of cotton, a leading natural fiber crop, is as old as the human ancient civilization. Traces of cotton (∼7000 years old) have been recovered from archaeological sites in Mexico. Also, the cultivation of desi cotton (*Gossypium arboreum* L.) in Mohenjo-daro (located in Sindh, Pakistan) was dated back to 6000 BC (Moulherat et al., 2002). Similarly, the evidence of cotton usage around 1500 BC was also reported in Reg Vida—the most ancient text of Sanskrit. Cotton cloths were introduced to Europe about 800 AD by Arab merchants. Later, the revolution in textile industry (spinning, weaving, etc.) not only paved the way for the introduction of cotton genotypes worldwide at massive scale but also replaced the cultivated diploid species (*G. arboreum* and *G. herbaceum*, both carry A-genome) with tetraploid cotton (*G. hirsutum*) in most parts of the world including India and Pakistan. It is worth to mention that *G*. *hirsutum* L. was introduced in subcontinent (India, Pakistan, and Bangladesh) ∼200 years ago, however, its cultivation started on a large scale in the 1930s in parallel to the revolution in textile industry (Rahman et al., 2014c). Now, cotton is cultivated in more than 80 countries—cultivated on 32.6 million ha with an annual production of 24.1 million tons^1^; however, the major cotton growing countries are China, India, United States, Pakistan, and Uzbekistan. Presently, cotton production is stagnant or even decreasing in many parts of the world due to the prevalence of multiple biotic and abiotic stresses. Among the biotic stresses, cotton leaf curl disease (CLCuD) is one of the major growing threats to cotton production. Historically, a first evidence of CLCuD was reported on native cultivated cotton species (*G. peruvianum* and *G. vitiforum*) in Nigeria in 1912 (Kirkpatrick, 1931). The word “leaf curl” was coined because of the typical upward curling of the leaves. In 1924, it was noticed for the first time on *G. hirsutum* in Oyo, Nigeria with characteristic symptoms of downward curling of leaves and change in leaf texture and color. The disease also infected the cotton crop in Sudan in 1924, followed by the outbreak of the disease in cotton fields of Northern Africa and Tanzania (Hussain et al., 1991). It took almost four decades to reach Pakistan. It was reported for the first time in a village near Multan, Pakistan in 1967, but remained neglected until it appeared in epidemic form in the early 1990s (Briddon and Markham, 2001). The total financial losses occurred between 1992 and 1997 were ∼5 billion US$ to the economy of Pakistan (Idris, 1990). The disease was also transmitted to cotton crop in India (Chowda Reddy et al., 2005) which was grown at the periphery of cotton growing belt of Pakistani Punjab, and later spread to the northern cotton growing areas of India (**Figure 1**).

**FIGURE 2:**
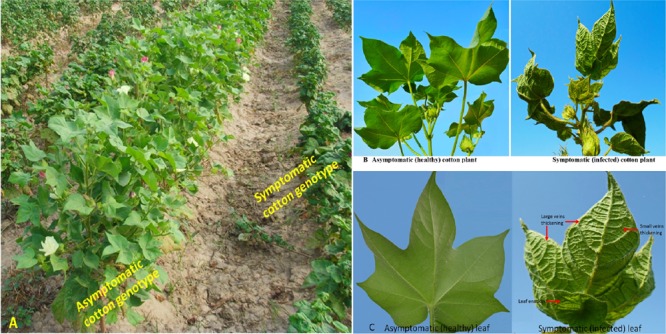
Comparison of symptoms of healthy and infected cotton plants. **(A)** Field view of asymptomatic (middle) and symptomatic cotton genotypes. **(B)** Asymptomatic versus symptomatic cotton plants. **(C)** Comparison between healthy and infected cotton leaf. Thickening of veins, curling of leaves and formation of leaf enation can be observed at the underside of the infected leaf.

Cotton leaf curl disease is characterized by small and large veins thickening and upward or downward curling of the leaves. Severe disease infection results in the development of leaf enation and finally retards growth of the cotton plant which substantially reduces cotton yield by 15–70% (Idris, 1990; Brown, 2001) (**Figures 2A–C**). This disease is caused by a complex of geminiviruses—transmitted through whitefly (Brown, 1992). According to the new classification, five different species: cotton leaf curl Multan virus (CLCuMuV); cotton leaf curl Bangalore virus (CLCuBaV); cotton leaf curl Kokharan virus (CLCuKoV); cotton leaf curl Allahabad virus (CLCuAlV); and cotton leaf curl Gezira virus (CLCuGeV) are responsible for causing the disease in different parts of the world (Muhire et al., 2014). Recently, CLCuMuV has been reported in southeastern China (Cai et al., 2010) and the Philippines—largely spread through the cross-border movement of traders carrying infected samples (cutting of hibiscus plants, etc.) from Pakistan to China. Whitefly is responsible for transmission of the virus locally; however, its role in transmitting virus from Pakistan to China or in other countries has not yet been established (Ashfaq et al., 2014). Recently, isolates of CLCuMuV collected from the Philippines and China were grouped into a cluster of the isolates reported from Pakistan. The Philippines is geographically isolated from other countries of Asia where CLCuMuV is present. Thus it is suggested that infected cuttings of hibiscus introduced in the Philippines are the primary source of virus transmission.

The virus complex is still evolving. A substantial variation in the viral genome sequence has been found in Pakistan (Saleem et al., 2016). Secondly, recombination between two major group of viruses, CLCuMuV and CLCuKoV resulted in the evolution of new viruses. Thus sustainability of cotton production is at potential risk in many cotton growing countries.

Cotton leaf crumple disease (CLCrD), another viral disease containing bipartite genome, is also transmitted by *Bemisia tabaci*. This disease substantially depressed cotton production in United States and Mexico in mid of the 20th century (Dickson et al., 1954). Typical symptoms appear on infected cotton plants are leaf crumpling and discoloration, and also reduced internodal length (Dickson et al., 1954). Later, the disease was reported on cotton plants—showing unique mosaic, in Guatemala (Brown, 2002). The disease was also found on other plant species including zucchini, watermelon (Akad et al., 2008), common beans (Adkins et al., 2009), etc. The disease is extremely damaging if infected the cotton seedlings before reaching the age of 10–14-leaf stage. This disease was controlled by reducing the whitefly population in the field. Breeders also identified cotton cultivar Cedix as resistant to CLCrV which was extensively used to develop tolerant cotton lines (Wilson and Brown, 1991).

In Pakistan, resistant cotton varieties were developed by crossing resistant sources (LRA 5166, Cedix and CP15/2) with local cultivated susceptible varieties. The resistance in the newly released cotton varieties remained intact until the emergence of a Burewala viral strain of the disease—appeared in Vehari District of Punjab in 2001. All resistant cotton varieties got infected with the newly evolved resistant breaking strain. This strain was named cotton leaf curl Burewala virus (CLCuBuV). This strain was also reported in India, and replaced the old strains of CLCuRV and CLCuKoV from the cotton fields of India (Rajagopalan et al., 2012). Thus cotton crop grown in India, China and Pakistan (contributes together >60% of the world cotton production) are at potential risk to CLCuD—highlighting the need for undertaking control measures to control this disease in all cotton growing regions worldwide. Currently, no cotton variety is resistant to CLCuD. Efforts are underway to develop transgenic cotton plants exhibiting high resistance to the disease (Malik et al., 2016; Wang et al., 2016; Vyas et al., 2017).

## Causal Organisms of CLCuD

The nature of the causal agents of CLCuD was described for the first time in 1926 (Jones and Mason, 1926). The vector of the disease was identified and named whitefly (*Bemisia* species, Golding, 1930). One year later, *B. tabaci* species was confirmed as the vector responsible for the transmission of the disease (Kirkpatrick, 1931). *B. tabaci* is a genetically diverse species, which is not only a source of virus transmission but also retards the growth of cotton plant by direct feeding (Jones, 2003; Incubar and Gerling, 2008).

Whitefly, a polyphagous insect can infest multiple hundred plant species (Williams, 2014). It suppresses the growth of infested plant by sucking the phloem sap. Whitefly is also a vector of nearly 200 plant viruses (Stewart et al., 2011). There are at least 24 biotypes of *B*. *tabaci* complex, identified using molecular as well as biological characteristics (De Barro et al., 2011). These species and or their variants are frequently found as a mixed population in natural environment (Legg et al., 2014). However, fluctuation in response for their preference to host as well as capabilities to transmit virus have been demonstrated (Legg et al., 2014; Polston et al., 2014). Recently, the *B. tabaci* complex has been sequenced using next-generation sequencing (NGS) in several laboratories (Wang et al., 2012; Ye et al., 2014; Zhu et al., 2016) which would help in resolving the phylogenies of the whitefly complex as well as studying their interaction with the host.

**FIGURE 3:**
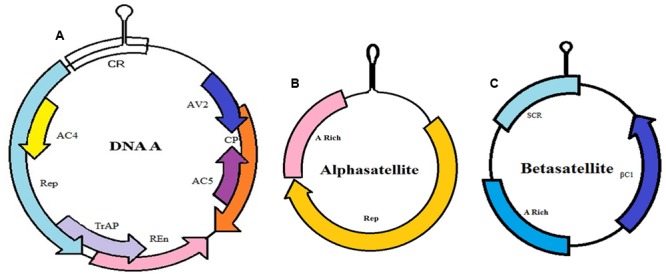
Complex of CLCuD. **(A)** Genome organization of begomovirus monopartite DNA A. This component encodes for replication-associated protein (Rep), coat protein (CP), replication enhancer protein (REn), transcriptional activator protein (TrAP), proteins involved in virus movement (AV2), pathogenicity determinant and a suppressor of RNA silencing (AC4) and proteins for viral genome replication (AC5). **(B)** Alphasatellites contain A-rich region and large region encode for Rep protein (Rep). **(C)** Betasatellites have a βC1 gene, satellite conserved region (SCR)—conserved among all betasatellites and A-rich region).

The CLCuD is caused by a group of begomoviruses (family *Geminiviridae*). However, a complex comprising of a monopartite begomovirus DNA A, betasatellite and alphasatellite, are responsible for causing the disease (**Figure 3**). The genomic size of the begomovirus is 2.8 kb. It consists of several genes encoding for replication-associated protein (Rep), coat protein (CP), replication enhancer protein (REn), transcriptional activator protein (TrAP), proteins for virus movement (AV2), proteins for pathogenicity determination and a suppressor of RNA silencing (AC4) and viral genome replication (AC5) (Briddon et al., 2001, 2002; Amrao et al., 2010).

Symbiotic relationship exists among these molecules, which forms one complex due to the interaction of proteins produced by these molecules. Alphasatellites, consisting of ssDNA molecules, do not exhibit any significant sequence identity with the helper virus (Saunders et al., 2000), have the capability to replicate autonomously. The size of this molecule is about 1.4 kb having a hairpin structure containing TAGTATTAC that forms an origin of virion-strand. These molecules encode a single protein called replication associated protein (Rep) that is more genetically similar with the Rep encoded by nanoviruses—transmitted by aphids. However, alphasatellites are dependent upon the helper viruses for their transmission in vector (whitefly) as well as their movement in plant. Their precise function has not yet been established (Idris and Brown, 2002; Xie et al., 2010; Hameed et al., 2014). In another study, the role of alphasatellites in disease severity by impacting the virulence of helper virus has been elucidated (Idris et al., 2011).

Betasatellites are single stranded DNA molecules, each ∼1.4 kb. It requires a monopartite begomovirus for its replication and encapsidation. These molecules are highly conserved in structure, and contain βC1 (single coding gene), satellite conserved region (SCR) and A-rich region. The βC1 gene appears to determine the symptoms of the disease (Iqbal et al., 2012). These molecules have the capabilities to interact with the helper viruses infecting a number of plant species including cotton (Xie et al., 2010; Briddon et al., 2014; Sartaj et al., 2014). These molecules share very little similarity with DNA-A begomoviruses except for a conserved hairpin structure (Akhtar et al., 2014).

### Detection and Identification of CLCuD and Its Viral Causal Agents

Knowledge of the mutations occurring in viruses is important for devising proactive breeding strategies to control the viral diseases. The viruses causing CLCuD are usually amplified by PCR using degenerate primers or specific primers. Another amplification method ‘rolling circle amplification’ (RCA) has been used to amplify multiple helper viruses as well as their recombinants (Inoue-Nagata et al., 2004; Haible et al., 2006). Also the primers for detecting the associated satellites molecules are available (Amrao et al., 2010; Idris et al., 2011). However, user friendly identification assays have not yet been evolved. In future, with the advancement in genome sequencing tools, it would be possible to design assays which could be used to detect and identify the whole complex including vector and virus complex in the field as well as monitoring the spread to other multiple crop species (Saleem et al., 2016).

## Strategies for Controlling the Disease

The CLCuD is a threat to all cotton growing countries where whitefly is prevalent as minor or major pest. A number of short term (mainly management practices) and long term strategies (developing resistant cotton varieties) were devised to control this disease. Long term strategies involve the development of resistant cotton cultivars either through conventional or non-conventional means.

### Short Term Strategies

After the spread of the disease at a massive scale in Pakistan, a number of short-term strategies were developed to control this disease by reducing the vector population in the field. For example, seed treatment for avoiding the early establishment of whitefly populations, control of whitefly using pesticides on cotton crop, eradication of weeds (alternative host for virus), better health of the plant through providing balance dose of fertilizers, biological agents, etc. were the immediate measures taken to control this disease (Narula et al., 1999; Cook et al., 2011; Cuthbertson et al., 2011; Yuan et al., 2012; Basit et al., 2013; Smalling et al., 2013; Horowitz and Ishaaya, 2014; Hollis, 2015; Huseth et al., 2016; Follett, 2017).

The recommendations for managing the size and density of the whitefly population are based on certain threshold level (application of chemicals is recommended at 4–5 whitefly per leaf in Pakistan irrespective of the fact that a single viruliferous whitefly can transmit virus from one plant to the other). Success for controlling the whitefly population was demonstrated to a certain degree by the application of selective insecticides—not only safeguarding the population of predators but also delaying the process of development of resistance to chemicals in whitefly population (Yuan et al., 2012; Roditakis et al., 2017). The resistance can also be delayed by using a different class of compounds with different mode of action (Ellsworth et al., 2006; Basit et al., 2013; Horowitz and Ishaaya, 2014).

In Pakistan, it is advisable to restrict the whitefly population size on cotton crop from its emergence till 70–90 days after sowing (DAS). In this regard, growers always prefer to treat seed with insecticides which provide protection from whitefly infestation up to 45 DAS or in some reports up to 75 DAS (Singh et al., 2002) followed by spraying chemicals. The effectiveness of this method to control the disease is largely based on protecting cotton plants from whitefly infestation up to 70–90 DAS. After this time period, the cotton plant is old enough to escape the disease, thus the losses can be minimized. The application of bio pesticides is another control measure to control the whitefly population (Sarwar and Sattar, 2016), however, its impact is yet to be realized.

The CLCuD is not seed-borne. It survives on alternative hosts such as tomato, tobacco, hibiscus, okra, and ageratum. Leaf curl-like symptoms have been observed on many herbaceous and woody species in the field including cotton, okra, hibiscus, hemp, sunflower, tobacco, and many weeds (Nour and Nour, 1964). Weeds may help in maintaining the reservoirs of inoculums of viruses as has been reported for the tomato yellow leaf curl virus (TYLCV) (Cohen et al., 1988; Ghanim, 2014). In general, removal of weeds from cotton crop reduces the chances of availability of alternate hosts—thus minimizing the potential sources of inoculum. In developing countries like India and Pakistan, farmers usually grow vegetables, oil seed crops, fodders, etc. (alterative hosts for the virus) in vicinity of the cotton field—further complicating the situation for taking effective control measures in controlling the vector as well as the disease. These alternative hosts provide a congenial condition to these viruses for making genetic recombination with other viruses infecting the host plants. Such recombinations often evolve new strains of the viruses. Thus banning of cultivation of alternative hosts in the close vicinity of cotton crop would help in controlling the vector population. Similarly, avoiding the cultivation of other crops in off season in cotton-growing areas may help in breaking the lifecycle of whitefly (Rafiq et al., 2008; Knight et al., 2017). Clean cultivation is important for controlling the whitefly population and disease incidence either through cultural practices or by the application of weedicides. Similarly, the plant secondary metabolites (for example repellents) have been reported for providing certain degree of protection against whitefly infestation. Intercropping of highly aromatic plants such as basil and coriander protects tomato from the infestation of *B. tabaci* (Carvalho et al., 2017).

Damage of CLCuD can also be minimized by the application of appropriate dose of fertilizers. For example, use of potassium can boost resistance to diseases possibly due its role in osmoregulation, synthesis of molecular compounds and in maintaining energy gradient. It also affects the compatibility relationship of host-parasite by impacting on the metabolic function (Kafkafi et al., 2001)—thus may help in controlling the CLCuD. In contrary to this, the use of excessive quantity of nitrogenous fertilizers reduces the disease resistance. It is suggested that a balance ratio of nitrogen and potassium fertilizer can help in reducing the disease severity.

It is concluded that controlling the whitefly populations by chemical, cultural, and biological means, complete elimination of the disease is not possible due to the fact that a small whitefly population may result in disease transmission hence leading to disease incidence.

### Long Term Strategies

#### Genetics of Resistance to CLCuD

Breeding of virus-resistant cotton varieties with sufficient genetic diversity has been suggested as a durable strategy for controlling the disease effectively (Rahman et al., 2002, 2005). Before developing virus resistance cotton varieties, genetic basis of resistance and its inheritance are the key components for designing breeding strategies (Hutchinson and Knight, 1950). Little is known about the molecular basis of resistance. Usually, multiple plant species have developed defense mechanism in a period of ∼350 million years for controlling insect pathogen and disease (Gatehouse, 2002). For example, a constitutive defense system, physical barrier (i.e., thickness and trichomes), synthesis of secondary metabolites (glucosinolates, alkaloids, gossypol, cyanogenic glucosides, phenolics, and proteinase inhibitors) and toxic compounds have been evolved (Arimura et al., 2011; Furstenberg-Hagg et al., 2013). In a recent study, cotton infested with whitefly and aphid showed variation in expression of transcripts associated with sugar and amino acid metabolism (Dubey et al., 2013). Furthermore, WRKY40 (a transcription factor) and copper transport protein may regulate cotton defense for controlling whitefly infestation. Silencing GhMPK3 (mitogen-activated protein kinase in *G. hirsutum*) by virus-induced gene silencing (VIGS) resulted in suppression of the MPK (mitogen-activated protein kinase)-WRKY-JA (jasmonic acid) and ET (ethylene) pathways which enhanced the whitefly susceptibility. Thus these genes can be used in developing host plant resistance (Li et al., 2016).

Earlier, resistance to the viral causal agents of CLCuD was assumed to be unstable because of several environmetnal factors including temperature, relative humidity, light, plant age, etc., may affect the disease incidence and severity (Rahman et al., 2005). It has been reported that resistance is conferred by a major gene (Knight, 1948). In another comprehensive study, two dominant resistant genes (R1 CLCuDhir and R2 CLCuDhir) and one suppressor gene were reported (S CLCuDhir) (Rahman et al., 2005). Also, two genes conferring resistance to the viral causal agents and disease was reported in 2007 (Ahuja et al., 2007). Tolerance to CLCuBuV has been recognized as a complex trait with incomplete expression.

#### Development of Resistant Cotton Varieties Using Host Plant Resistance

Breeding of resistant cotton varieties is the only effective way for controlling the disease and its viral causal agents, particularly when infection occurs early and routinely during the season. The variability in incidence and severity of the disease depends upon the host genetic makeup, virus titer and severity of the disease, and whitefly population (Baluch, 2007).

A number of virus and disease resistant cotton varieties were developed using recombination breeding approaches. Resistant sources were identified by screening more than 1000 cotton genotypes/accessions available in the gene pool of CCRI Multan (Muhammad Afzal, CCRI Multan, personnel communication) in hot spots under natural conditions. Three cotton genotypes (‘LRA-5166,’ ‘CP-15/2,’ and ‘Cedix’) were found resistant. However, ‘LRA-5166’ and ‘CP-15/2’ were used extensively for deriving resistance into the cultivated susceptible cotton cultivars through various hybridization breeding procedures.‘CIM-1100’ was the first resistant cotton variety released from CCRI Multan in 1997 followed by a series of resistant cotton varieties (‘MNH-552,’ ‘CIM-448,’ ‘CIM-496,’ ‘NIBGE-2,’ ‘FH-901,’ etc.) developed by CCRI Multan and other cotton breeding research institutes (Rahman and Zafar, 2007; Arshad et al., 2009; Rahman et al., 2014c).

Deployment of these sources of disease resistance resulted in narrow genetic base (Rahman et al., 2014c). This resistance was overcome within 5 years by the evolution of the Burewala strain. Till today, none of the variety was found completely asymptomatic. However, tolerant cotton genotypes, viz. ‘NIBGE-115’ (Rahman and Zafar, 2007), ‘FH-142,’ and ‘NN-3’ (Rahman and Zafar, 2012) have been identified which can control the disease.

Multiple efforts were made to identify resistant sources by screening the material under controlled conditions (through grafting of infected buds) and/or natural conditions by exposing the cotton plants with viruliferous whiteflies (Rahman et al., 2002). However, for massive screening of cotton germplasm, screening under field condition is more practical. In this regard ∼5000 accessions of *G. hirsutum* L. and introgressed lines were introduced from the United States Department for Agriculture (USDA) in Pakistan to screen the material against CLCuBuV/disease. Initial studies have shown that ‘Mac-07’ and the other 95 cotton accessions were found asymptomatic to the disease (Rahman et al., 2014b). However, most of these resistant genotypes are photoperiod sensitive. These newly identified sources can be used extensively in improving the cotton germplasm/varieties resistant to the CLCuD and its viral causal agents.

**FIGURE 4:**
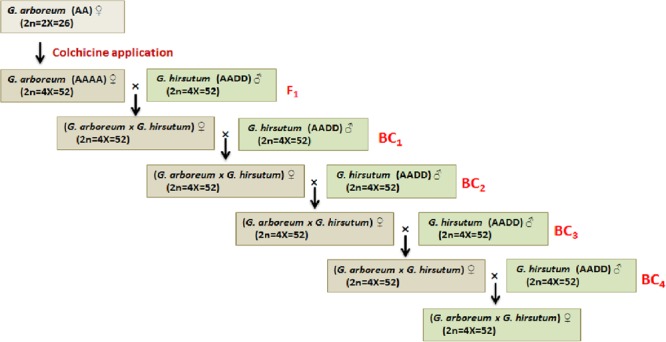
Schematic diagram showing the process of gene introgression from *G. arboreum* (highly resistant/immune to the viral causal agents of CLCuD) into *G. hirsutum* (susceptible to disease).

Diploid cotton (*G. arboreum* and *G. herbaceum*) grown in Asia and Africa prior to the introduction of tetraploid cottons (*G. hirsutum* and *G. barbadense*), is resistant and/or immune to CLCuD and its viral causal agents (Rahman et al., 2005). *G. robinsonii* has been identified as a new resistant species (Azhar et al., 2011). A number of crosses between *G. arboreum* and *G. hirsutum* were made, however, success rate toward the development of resistant varieties was limited because of the linkage drags of unwanted traits coming from *G. arboreum*. Alternatively, introgression from *G. hirsutum* traits into *G. arboreum* (hirsutization of *G. arboreum—*introgression of economically important traits from *G. hirsutum* into *G. arboreum* background) was carried out but none of the variety was developed using this technique (**Figure 4**). In these experiments, the chromosome number of *G. arboreum* was doubled by the application of colchicines followed by hybridization with the allotetraploid *G. hirsutum* under natural conditions. All the F_1_’s exhibited resistance to the CLCuD and its viral causal agents after graft inoculation with infected buds (Ahmad et al., 2011). The successive backcross generations got little symptoms but still were more tolerant than the cultivated standard variety (CIM-496). For example, disease incidence was 1.7–2.0%, 1.8–4.0%, and 4.2–7.0% in BC_1_, BC_2_, and BC_3_, respectively, which was less than ‘CIM-496’ (96%). Cytological studies of CLCuD resistant plants revealed that the frequency of univalents and multivalents were high in BC_1_ but low in BC_2_. In BC_3_, substantial number of plants retained significant number of bolls because of the high frequency of chromosome pairings (bivalents). Thus, this study has shown successful introgression of resistant genes from *G. arboreum* to *G. hirsutum* (Nazeer et al., 2014).

#### Use of Genetic Approaches for Improving Resistance to CLCuD and Its Viral Causal Agents

Use of various genetic tools is essential in developing genetic linkage maps, tagging genes of interest, determining gene function, regulation and their expression, and in developing transgenic plants. In linkage mapping, genetic distances between a trait and DNA marker is estimated. The identified DNA markers can be used in initiating marker-assisted selection (MAS).

Use of DNA markers in developing CLCuD and its viral causal agents resistant cotton cultivars is extremely important because of several reasons. Firstly, field screening is time consuming as it is heavily dependent upon the vector population—fluctuates substantially due to the prevailing environmental conditions. Secondly, sources of virus inoculum (weeds, alternative hosts, etc.) may also impact the response of cotton genotype toward the disease and its viral causal agents. Local temperature and humidity further complicate the situation. For example, plants near the border of the cotton field will show different response than those plants of the same genotype growing inside of the field. Similarly, if cotton crop is planted near to orchards or gardens (makes the local environment more humid) would be more prone to the disease than that of the cotton field located distantly away from the orchards or gardens. Imposing uniform disease stress in greenhouses is extremely difficult due to fluctuations in the prevailing microclimate—may impact the screening procedure. Thus, use of DNA markers is more desirable to breed for true resistant cotton varieties.

Efforts were made toward the identification of DNA markers in Pakistan. However, limited genetic diversity among the genetic material was the major handicap in identification of robust DNA markers. For example, genetic similarity among the cotton cultivars released before the onset of the first epidemic of the disease and its viral causal agents ranged from 81.5 to 93.41% (Iqbal et al., 1997). The genetic similarity among the cotton cultivars-genotypes released after the first epidemic of the disease was in the range of 81.45 to 94.90% (Rahman et al., 2002).

Many cotton researchers have conducted studies to map CLCuD and its viral causal agents resistance QTLs using intra- and inter-specific crosses. RFLP markers were used to identify DNA markers associated with resistance to the disease. An F_2_ population was evaluated using RFLP. A total of three DNA marker loci linked to resistant loci were identified (Aslam et al., 1999). In another study, random amplified polymorphic DNA (RAPD) assay was deployed to identify markers linked to genes conferring resistance to the disease. A bi-parental F_2_ mapping population was derived using *G. hirsutum* var S-12 (as susceptible source) and *G. hirsutum* var LRA-5166 (as resistant source). Bulked segregant analysis (BSA) was deployed by pooling equal quantity of genomic DNA of resistant and susceptible F_2_ plants in two different pools. In total, 520 decamer random primers were surveyed on these bulks. Unfortunately, polymorphic RAPD primer was not identified. Then these RAPD primers were surveyed on the parental genotypes of experimental population. A total of 13% of the amplicons were polymorphic. A RAPD marker was identified in *trans* phase with 14% recombination frequency. While OPO-19_460,_ OPQ-14_325_, and OPY-2_1080_ (in coupling phase with 0–5% recombination frequency) were found associated with resistance to the disease (Rahman et al., 2002, 2005). In another study, a total of 18 cotton genotypes were screened for CLCuD and its viral causal agents. Only two genotypes CIM-240 and CIM-442 showed resistance against the disease and its viral causal agents (Mumtaz et al., 2010).

In one of the earlier studies, intraspecific F_2_ population of *G. hirsutum* was developed by crossing LRA-5166 (resistant) and S-12 (susceptible). This F_2_ population was screened with RAPD, SSRs, and AFLP primers. A total of 225 RAPD primers were screened on two parents. In total, 11 were found polymorphic among the parents. These polymorphic primers were surveyed on F_2_ population to find their association with disease resistance. Out of these, three marker loci (OPO-19, OPQ-14, and OPY-2) were linked with the resistance. A total of 34 (out of 215 SSRs) were found polymorphic among the parent genotypes. Only JESPR-151 showed association with resistance to CLCuD and its viral causal agents (Niaz, 2005).

Efforts for the identification of DNA markers associated with resistance to CLCuBuV and its disease started in 2012. A mapping population was developed by crossing a highly tolerant genotype var. 2472-3 of *G. hirsutum* with the highly sensitive genotype var PGMB-66 of *G. barbadense* (Rahman et al., 2014a). A total of 2400 SSRs were initially selected to explore the genomes of both species. Out of these, 113 SSRs were found polymorphic and subjected further to screen the F_2_ population. In these preliminary studies, two QTLs, i.e., QCLCuD25 and QCLCuD26 associated with CLCuD resistance were identified. In order to construct a high resolution genetic linkage map, more SSRs are needed to survey the F_2_ population (Rahman et al., 2014b). In another study, a total of 10 cotton genotypes (five highly tolerant, four highly susceptible, and one immune) were selected out of 1200 cotton genotypes (screened for two seasons). A total of 322 SSR primer pairs derived from bacterial artificial chromosome (BAC) end sequences of *Gossypium raimondii* were surveyed on the selected cotton genotypes. Out of these, 65 primer pairs were found polymorphic, and the studied genotypes were grouped into two distinct clusters comprising of tolerant and susceptible genotypes, respectively. Among the polymorphic markers, two SSR markers, PR-91 and CM-43 were amplified only in tolerant genotypes which showed significant association with resistance to CLCuD and its viral causal agents (Abbas et al., 2015).

#### Use of Transgenic Approaches

Multiples strategies have been deployed to develop cotton plant conferring resistance to CLCuD and its viral causal agents using genetic engineering approach. These strategies are largely based on using different small conserved portion or full length genes of the virus (pathogen derived resistance), and genes from other distantly related genetic sources (non-pathogen derived).

##### Pathogen derived resistance (PDR)

Introduction of a part of virus genome (gene or part of a gene) which is usually conserved across several virus genomes of the same species has been considered as the most useful strategy for controlling the diseases (Goldbach et al., 2003). Following strategies which have been used to develop resistance using the genome information of the viruses.

##### Antisense RNA technology

This technology works by silencing the complementary target mRNA by the antisense RNA molecule—thus inhibiting the expression of the target mRNA. A study was conducted for targeting the *rep* gene of virus in transgenic cotton which suppressed the replication of the invading virus (Amudha et al., 2010). In another study, ACP gene (AV1) was targeted for arresting viral replication, movement and encapsidation in transgenic cotton (Amudha et al., 2011).

Transgenic cotton was developed in an Indian variety (‘F846’) via Agrobacterium-mediated transformation using antisense movement protein gene (AV2). A binary vector pPZP carrying the antisense AV2 (350 bp) gene along with the *NPTII* gene was used. Transgenic nature of the putative transgenics was confirmed by conducting molecular analysis, and these plants were found to be resistant against CLCuD and its viral causal agents (Sanjaya et al., 2005).

Similarly, two truncated forms of replicase (tACI) gene was introduced in *G. hirsutum* for inhibiting the replication of viral genome and β satellites DNA components (Hashmi et al., 2011). Transgenic cotton (*G. hirsutum* cv. Coker 310) was also developed by introducing βC1 gene in antisense orientation under 35S promoter. Successful introduction of the gene in cotton genome was confirmed by Southern blot hybridization. It has been demonstrated that the transgenic cotton remained symptomless (Sohrab et al., 2014). In multiple reports, the transformation efficiency using *A. tumefaciens* mediated transformation was calculated about 0.3% (Amudha et al., 2011; Hashmi et al., 2011).

##### RNAi

Principally, RNAi is based on the post-transcriptional gene silencing (PTGS) and transcriptional gene silencing (TGS), and it was deployed for studying function of genes. It has also been applied to develop resistance to viral diseases (Tenllado et al., 2004; Khalid et al., 2017) in multiple crop species. For instance, it has been used to develop resistance to African cassava mosaic virus (ACMV) (Chellappan et al., 2004), mung bean yellow mosaic virus (MYMV) (Pooggin et al., 2003) and several others. A 21 nt long sequence of V2 gene of CLCuBuV was used to make artificial microRNA (amiRNA) constructs followed by testing response in a model species *Nicotiana benthamiana*. The transgenic plants were found asymptomatic when challenged with CLCuBuV. It was also concluded that the magnitude of resistance is based on the extent of complementarities between amiRNA and the target sequence, and also the sequence of miRNA backbone (Ali et al., 2011). Recently, the RNAi-based construct targeting the V2 gene of CLCuKoV-Bur was transformed using apex cut method in two cotton cultivars MNH-786 and VH-289. Copy number of the transgene and its location was spotted using FISH and karyotyping analysis of T2 generation. The gene was integrated on chromosome number 6 and 16. In the stable transgenic lines, low titer of the virus was reported when challenging the cotton plants with whitefly under contained conditions. From the results, it was concluded that amplicon V2 RNAi construct was able to limit virus replication and can be used to control CLCuV in the field (Yasmeen et al., 2016).

##### Plant host enzymes and hormones

Interaction of host proteins with the viruses is a well-established phenomenon. These interactions lead to suppress the host protein gene or otherwise. It has been proved that the βC1 protein gene—a pathogenicity determinant, of satellite β DNA (associated with CLCuMuV) interacts with the host Ubiquitin-conjugating (E2) enzyme S1UBC3 (Eini et al., 2009). In this interaction, the overexpression of βC1 in transgenic plants suppressed the accumulation level of polyubiquitinated proteins. It has also been reported that this interaction is correlated with disease severity (Bachmair et al., 1990). Further experiments are needed to exploit such interactions for controlling the invading virus in cotton.

In plants, jasmonic acid, a major defense hormone, is effective to control herbivorous insects and necrotrophic pathogens. In a recent study, its role in conferring resistance to insects in young plants of Arabidopsis has been demonstrated, and is regulated by miR156-targeted-SPL9 (negatively correlated with JA expression) (Mao et al., 2017). Such novel pathways can be exploited in cotton for controlling the whitefly and other chewing insect pests.

##### Non-pathogen derived resistance

Genes from host or non-host plant rather than the causal agent are used to engineer resistance against the disease. For example, genes responsible for conferring DNA binding proteins, coat binding proteins, antiviral antibodies, etc., have been introduced in plants to induce resistance to CLCuD and its viral causal agents (Lomonossoff, 1995; Castellano et al., 1999). Recently, a mechanism that confers resistance to phytophagous insects in ferns and mosses has been explored. For example, a protein Tma12 was identified in fern which confers resistance to whitefly. The gene encoding this protein was transformed in cotton Coker 312. Out of many, one transgenic cotton line has shown increased resistance (>99%) to whitefly. This protein has been found non-toxic in rats. Thus this gene can be used in future for controlling whitefly in other crop species (Shukla et al., 2016).

##### DNA binding proteins AZP

Virus resistance was developed through transgenically expressed DNA binding proteins. These have been designed in such a manner that these will not bind to host DNA sequences. For example, Rep, a sequence-specific dsDNA binding protein (Castellano et al., 1999), binds to direct repeats in the virion strand (v-ori), and thus inhibits the viral replication (Fontes et al., 1992). Subsequently, artificial zinc finger (AZP) proteins were designed to target Rep-specific direct repeats of the v-ori of the invading geminiviruses (Sera and Uranga, 2002). Use of this technology was successfully demonstrated in sunflower, rice, wheat, etc., where the resistant genes contain multiple ZF domains (Gupta et al., 2012). However, demonstration of this technology for controlling the geminiviruses in cotton is yet to be proved. TALEN, a genome editing tool, which could be deployed as an alternative to AZP. The TALEN comprises of non-specific FokI nuclease domain that is fused to a customizable DNA-binding domain and DNA-binding domain—contains repeats of conserved nature which are derived from transcription activator-like effector proteins (TALEs). The TALEs has the capability to change the gene transcription in host cell (Khan et al., 2017). This phenomenon can also be used for controlling the CLCuD and its viral causal agents.

##### GroEL-mediated protection

The GroEL protein, produced by a bacterium residing in the gut of whitefly, binds to a coat protein of begomoviruses, resultantly these viruses can be destroyed in the hemolymph of whitefly (Morin et al., 2000; Rana et al., 2012). Resistances to a number of viruses of different taxonomic genera can be developed using this tool (Edelbaum et al., 2009). For instance, *B. tabaci* GroEL gene expressed in transgenic tomato protected it from yellow leaf curl virus (TYLCV) (Akad et al., 2007). A relationship has been established between the GroEL protein and the transmission of potato leaf roll virus and TYLCV by aphid and *B. tabaci*, respectively (Kliot and Ghanim, 2013). These GroEL proteins may contribute toward adding resistance to multiple virus species as it has been elucidated by expressing the GroEL protein gene in *Nicotiana benthamiana* (Edelbaum et al., 2009; Gorovits et al., 2013). Potential of these proteins for controlling CLCuD and its viral causal agents needs to be tested.

##### Cell death induction

This approach has been utilized for restricting the multiplication of geminivirus in transgenic plants. It was obtained by the combined action of barnase and barstar proteins derived from *Bacillus amyloliquefaciens.* Barnase is a ribonuclease (RNase) and barstar inhibits the activity of barnase. If there is no geminivirus infection, the two transgenes should express at the same levels for avoiding production of the RNase. This approach was experimented in controlling the tomato leaf curl New Delhi virus (ToLCNDV) and the spread of the virus to other tissue was arrested (Vanderschuren et al., 2007). Recently, suppression of whitefly population in transgenic tobacco plant expressing the insecticidal genes under phloem promoter has been reported (Javaid et al., 2016). However, its potential in controlling geminiviruses in cotton is yet to be realized.

## Genome Editing Approach: Crispr/Cas Resistance Strategy

The CRISPR/Cas9 system with higher level of specificity derived the attention of scientists from all major fields of science, especially plant biologists, as a promising genome editing tool apart from zinc finger (ZFN) and Transcription activator-like effector nucleases (TALENs). Furthermore its potential in controlling begomoviruses can be explored due to robustness, wide adaptability and ease in engineering of this system (Iqbal et al., 2016).

The clustered regularly interspaced palindromic repeat (CRISPR)/CRISPR/Cas9 system confers immunity to the invading nucleic acid (plasmids or phages) in bacteria (Bikard et al., 2014). The invading DNA molecules are chopped down by the CRISPR spacers. The resultant molecules (20 nt long) are analog to the molecules generated by RNAi (Marraffini and Sontheimer, 2010). These are present in ∼40 and 90% of the sequenced bacterial genomes and sequenced archaea, respectively (Grissa et al., 2007).

The CRISPR/Cas9 system has been exploited in multiple complex organisms for editing genomes by delivering the Cas9 protein and guide RNAs in a cell. Through this technology, several loci can also be targeted using multiple sgRNAs (Cong et al., 2013). Resistance to geminiviruses has been developed using CRISPR. For example, Bean yellow dwarf virus (BeYDV) genome was mutated with the CRISPR–Cass system in bean, and thus reduced multiplication of the virus in the host resulted in reduced disease symptoms (Baltes et al., 2015). Similarly, reduction in disease symptoms of TYLCV was reported using CRISPR/Cas9 systems (Ali et al., 2015). Also, resistance to beet severe curly top virus was developed in *Nicotiana benthamiana* using a sgRNA–Cas9 constructs (Ji et al., 2015). It has been suggested that this system can be used to control geminiviruses as one of the options (Chaparro-Garcia et al., 2015; Zaidi et al., 2016, 2017). To the extent of our knowledge, few collaborative projects have been initiated to control CLCuD and its viral causal agents; however, its success is yet to be demonstrated.

## Future Outlook

The resistance to CLCuD has been largely derived from *G. hirsutum*. Thus relying on a single source of resistance is another worrying issue that may challenge the future cotton fiber security. It is extremely important that new resistance sources with different mechanisms should be identified followed by pyramiding them into a single genotype for developing durable resistant cotton varieties. In this regard, collaborative efforts are required aiming at the exchange of expertise and genetic material. For example, USDA shared about 5000 cotton accessions for screening to CLCuD in Pakistan. Out of these, dozens of asymptomatic accessions have been identified, and are being utilized in breeding programs as well as in developing mapping populations for identifying DNA markers linked with resistance to the disease. The information generated and genetic material generated through this venture is not only useful for Pakistan but also for the international cotton growing community. Thus, everyone gets the benefit of undertaking such collaborative projects in the form of response of the screened cotton germplasm, useful knowledge on genetics and genomics of resistance to the disease, experiences about the stress, etc.

One of the progenitors species contributing A-genome (*G. arboreum* and or *G. herbaceum*) is immune to the viral causal agents of CLCuD, however, the genetic potential of these species for developing resistance in cultivated cotton has not been fully utilized because of dragging of some unwanted traits. In spite of the fact that genome of each of *G. arboreum* and *G. herbaceum* has been sequenced but genetic maps involving these two species as one the parent genotypes are limited. For identifying QTLs associated with disease resistance, it is important to make interspecific crosses using susceptible diploid and or tetraploid species that would help in identifying new DNA markers. These DNA markers can be utilized in backcross breeding scheme to recover the genome of recurrent genotype (*G. hirsutum*) while retaining the desirable alleles of the donor genotype (diploid species). A number of NGS tools can also be deployed to develop high density genetic maps and also for cloning resistant genes followed by introducing them in *G. hirsutum* through transgenic approaches would help breeders to develop resistant cotton varieties.

In Pakistan (like many parts of the world), cotton varieties especially released after the first epidemic of the viral causal agents of CLCuD, have narrow genetic base. It has been demonstrated that the release of cotton varieties with sufficient genetic diversity can buffer the spread of diseases. In this regard, underutilized genetic resources (land races, obsolete varieties, old accessions, etc.) are useful genetic resources for developing genetically diverse cotton varieties. For achieving success in breeding, the deployment of genomic tools for “re-sequencing” of germplasm, old varieties and land races would help in identifying genetic variations (SNPs) linking with the functional diversity, where afterward DNA markers can be designed. Alternatively, if the resources are meager, exome capturing or sequencing the transcriptomes may help in detecting variations in genes. These variations can be utilized for enhancing the diversity among the cultivars.

New technologies for mutating genes using conventional as well as non-conventional approaches would be instrumental in developing resistant cotton cultivars. TILLING (Targeting Induced Local Lesions IN Genomes) is used for inducing mutations randomly. The resultant stable mutant lines can be re-sequenced and or exome regions can be sequenced for identifying mutations. The modern genetic tools like ZFNs and CRISPR–Cas9 can induce mutations in the target genes without disturbing the whole genome, can help in understanding mechanism of host–virus interaction as well as can also be used to eliminate the invading viruses. However, their potential is yet to be realized commercially. Exploitation of RNAi technology using various pathogen (virus) genes (REP, CP, V2, etc.) may help in controlling the disease. Efforts have been made to develop genetically engineered resistant cotton varieties but were not successful due to several reasons including high evolution rate of viral strains and screening of cotton material under controlled conditions instead of screening extensively under natural field condition. Similarly, vector population (whitefly) can also be controlled using sex lethal genes. In addition, the mechanisms of interaction of virus with vector and host plant should be understood to devise durable strategies for controlling the disease. The non-conventional resistance can be combined (pyramid) with the natural resistance in one genotype, which is considered one of the ways to counter the fast evolving viral genomes.

Lastly, awareness about the disease to farmers and researchers, development of technical expertise, implementation of quarantine measures in true spirit in airports for testing plant material (alternative hosts including ornamental plants), and cultivation of improved cotton varieties developed through bridging conventional and genetic approaches would be instrumental in overcoming this disease.

## Author Contributions

MR prepared the outlines and edited extensively primary and subsequent draft of the article; AK wrote the virology part; ZR wrote the host plant resistance section; MI wrote the genomic section; YZ conceived the basic idea and gave suggestions to improve the article. All authors read and approved the article.

## Conflict of Interest Statement

The authors declare that the research was conducted in the absence of any commercial or financial relationships that could be construed as a potential conflict of interest.

## Footnotes

1 www.icac.org/Press-Release/2017
